# PADI4 facilitates stem‐like properties and cisplatin resistance through upregulating PRMT2/IDs family in oesophageal squamous cell carcinoma

**DOI:** 10.1002/ctm2.70272

**Published:** 2025-03-13

**Authors:** Zeyu Wang, Hao Wu, Zhaoxing Li, Zhukai Chen, Anqi Feng, Yuan Chu, Kang Fang, Zehua Zhang, Ziying Zhao, Zhuyun Leng, Shihan Zhang, Xiaoyuan Wang, Lingnan He, Tao Chen, Meidong Xu

**Affiliations:** ^1^ Department of Gastroenterology Endoscopy Center Shanghai East Hospital School of Medicine Tongji University Shanghai China

**Keywords:** cisplatin, citrullination, OSCC, PADI4, PRMT2

## Abstract

**Background:**

Oesophageal squamous cell carcinoma (OSCC) is a highly lethal cancer characterized by its aggressive nature and chemotherapy resistance. Peptidylarginine deiminase 4 (PADI4) regulates protein citrullination and is associated with various cancer developments. The role of PADI4 in OSCC progression and chemoresistance remains unexplored.

**Methods:**

The protein interactions were conducted by immunoprecipitation assays. Quantitative real‐time PCR and western blotting were utilized to quantifyexpression levels in cancer cells. The stem‐like properties were assessed through spheroid growth assays and Cancer Stem Cells (CSCs) markers. Additionally, the resistance of cancer cells to cisplatin was evaluated using CCK8 assay.

**Results:**

This study shows that PADI4 promotes cellular stemness, contributing to the progression and chemoresistance of OSCC. Mechanistically, PADI4 facilitates the citrullination of protein arginine methyltransferase 2 (PRMT2), a process essential for the stabilization of PRMT2 expression and the enhancement of its function in promoting the transcription of IDs family (ID1 and ID2) via histone arginine methylation. This mechanism subsequently increases tumour stemness and contributes to the cisplatin resistance observed in OSCC. Mutations at the R312 site or inhibition by GSK484 can attenuate tumour stemness in OSCC, thereby reducing cisplatin resistance.

**Conclusion:**

PADI4 promotes citrullination and stabilization of PRMT2, enhancing its function in upregulating ID1 and ID2 expression via histone arginine methylation, which increases stemness and contributes to cisplatin resistance in OSCC; this effect can be mitigated by R312 mutations or GSK484 inhibition, reducing stemness and cisplatin resistance.

**Key points:**

- The role of citrullinization in cisplatin resistance of OSCC.
- PADI4 citrullinate of PRMT2 and stabilize PRMT2.
- PADI4 citrullinate of PRMT2 promoting the transcription of IDs family (ID1, ID2 and ID3) via histone arginine methylation.
- PADI4 citrullinated PRMT2 affected the combination of PRMT2 and USP7.
- PADI4 citrullinate of PRMT2 at R312 site.
- PADI4 inhibitor GSK484 can affect the stemness of OSCC and cisplatin resistance.

## Graphical abstract

Citrullinization is a post‐translational modification that has rarely been studied. Our research found that enzyme peptididylarginine deiminase 4 (PADI4) facilitates the citrullination of protein arginine methyltransferase 2 (PRMT2), a process essential for the stabilization of PRMT2 expression and the enhancement of its function in promoting the transcription of IDs family (ID1 and ID2) via histone arginine methylation. This mechanism subsequently increases cancer stem cells (CSCs) stemness and contributes to the cisplatin resistance observed in oesophageal squamous cell carcinoma (OSCC). Mutations at the R312 site or inhibition by GSK484 can attenuate stemness in OSCC, thereby reducing cisplatin resistance.

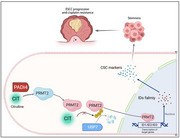

## 1 INTRODUCTION

Oesophageal cancer primarily includes two types: oesophageal squamous cell carcinoma (OSCC), responsible for approximately 90% of cases,^1^ and oesophageal adenocarcinoma, each with distinct epidemiological and pathological characteristics. OSCC is the sixth most common cause of cancer deaths globally, with over half of the cases occurring in China.^2^ Currently, few clinically validated methods exist for the early diagnosis and treatment of OSCC, resulting in the poor prognosis with a 5‐year survival rate under 20%.^3^ The statistics highlight the importance of early detection and treatment for OSCC. Exploring the molecular mechanisms of OSCC progression and identifying potential targets to hinder its advancement is crucial. In the current treatment of advanced oesophageal cancer, chemotherapy remains the most important therapeutic modality, with cisplatin being one of the key chemotherapeutic agents.^4^ Cisplatin resistance is a significant factor affecting the survival time of patients with advanced oesophageal cancer.^4^

Cancer stem cells (CSCs) are significant factors that refer to tumour‐initiating cell populations. Characterized by their ability to self‐renew and maintain a stem‐like state, CSCs, known for their self‐renewal and stem‐like properties, are pivotal in tumour initiation, maintenance, progression and drug resistance.^5^ Targeting CSCs‐associated signalling pathways offers a promising approach for addressing cancer, particularly in overcoming cisplatin resistance,^6^ especially in cisplatin resistance. Common markers for CSCs include CD144, CD44, Nanog, OCT4, SOX2 and others.^6^ In addition, several transcription factors previous identified as closely related to the regulation of stemness, such as ID1, ID2 and ID3.^7^, ^8^, ^9^

Global changes in epigenetics are considered a hallmark of cancer. During the formation and progression of tumours, post‐translational modifications of histones regulate complex gene expression networks, which in turn affect tumour growth, metastasis, and drug response.^10^ Peptidylarginine deiminases (PADIs) consist of five family members, namely PADI1‐4 and PADI6. All PADIs, except for PADI6 which is enzymatically inactive and primarily expressed in the ovaries, can modify positively charged arginine residues in substrate proteins into neutral citrulline residues through citrullination or deamination.^11^, ^12^ Recent evidence from both foundational and clinical studies supports the significant role of PADIs in the initiation and progression of cancer. Elevated PADIs expression in human cancers relative to healthy tissues, coupled with the efficacy of synthetic PADI inhibitors in eradicating various cancer cell lines, indicates that PADI‐catalysed citrullination may be pivotal in oncogenesis.^11^, ^13^, ^14^ The involvement of PADIs and their catalysed citrullination in OSCC development is not yet fully understood.

Protein arginine methyltransferases (PRMTs) facilitate the methylation of arginine residues in histone and non‐histone proteins as a form of post‐translational modification. The PRMT family, comprising PRMT1–PRMT9, generates three methylarginine types: asymmetric dimethylarginine, monomethylarginine (MMA) and symmetric dimethylarginine. PRMT family members are categorized into three types based on the methylarginines they produce: Enzymes classified as Type I (including PRMT1‐4, PRMT6 and PRMT8) enable MMA and asymmetric dimethylarginine modifications; Type II (PRMT5 and PRMT9) enable MMA and symmetric dimethylarginine; and Type III (PRMT7) is specific to MMA modification.^15^ Research has shown that histone arginine methylation influences both gene transcription and histone stability. The critical role of protein arginine methylation was highlighted in many studies in malignant tumour progression.^16^, ^17^ PRMT2, part of the PRMT family, catalyses the asymmetric dimethylation of H3R8 (H3R8me2a), thereby activating the expression of its target genes.^18^ Currently, no studies have investigated the role of PRMT2 in OSCC. Therefore, we are curious whether PRMT2 influence the malignant progression of OSCC.

This study investigates the mechanism of PADI4 in catalysing citrullination and its impact on OSCC progression, suggesting novel therapeutic strategies targeting PADI4 for OSCC treatment.

## 2 METHODS

### 2.1 Cell culture and reagents

Under standard conditions of 37°C and 5% CO_2_, the OSCC cells were grown in Dulbecco's modified Eagle medium (DMEM) with 10% foetal bovine serum (FBS). They were sourced from the Institute of Biochemistry and Cell Biology (Chinese Academy of Sciences).

### 2.2 Bioinformatics analysis

PADIs expression profiles in OSCC were examined using The Cancer Genome Atlas (TCGA) genomic data. Additional analysis was performed using publicly available datasets (GSE169337 and GSE23400) from the Gene Expression Omnibus (GEO) repository.

### 2.3 Clinical and specimens

Clinical specimens included 74 paired OSCC and adjacent non‐tumour tissues, as well as 98 OSCC samples with clinical records, obtained via surgical resection from treatment‐naive patients at Shanghai East Hospital, Tongji University. The study protocol was approved by the hospital's Ethics Committee (No. 2019061), with written informed consent from all patients. Tissues were paraffin‐embedded for immunohistochemical (IHC) analysis.

### 2.4 CCK8 assay

Cell proliferation and cisplatin sensitivity were evaluated using CCK8. Briefly, 1 × 10^3^ cells/well were put in 96‐well plates. After incubation, 10 µL CCK8 added, followed by a 2‐h reaction. Absorbance was measured using a microplate reader. Cisplatin‐treated cells were assessed after 48‐h exposure.

### 2.5 Spheroid growth assay

The assessment of three‐dimensional spheroid expansion was conducted as previously described in the literature. In summary, 1000 cells were plated as single‐cell suspensions in each well of a low‐attachment 6‐well plate using serum‐free DMEM/F12 (Gibco) with 2% B27 (Invitrogen), 20 ng/mL EGF (PeproTech) and 20 ng/mL bFGF (PeproTech). Spheroid growth was monitored over a period of 2 weeks using light microscopy.

### 2.6 Quantitative real‐time PCR

RNA was extracted by TRIzol from Invitrogen. The first cDNA strand was synthesized using HiScript II qRT Super Mix (Vazyme). Quantitative real‐time PCR was performed with qPCR SYBR Green PCR Master Mix (Yease) and gene primers listed in Table S1. The expression levels of all target genes were normalized using GAPDH or β‐actin as reference genes, and relative expression changes were determined by the 2^−ΔΔCT^ method.

### 2.7 Western blot

Cells were rinsed twice with cold phosphate‐buffered saline (PBS) before performing the analysis. Cells underwent lysis for half an hour in a chilled RIPA buffer, which included protease inhibitors and was made up of 50 mM Tris–HCl (pH adjusted to 7.4), 150 mM sodium chloride, 1% Triton X‐100, 1% sodium deoxycholate and 0.1% Sodium Dodecyl Sulfate (SDS). Supernatants were collected by centrifugation, and proteins were separated via SDS‐PAGE (10% or 15%) before transfer to Polyvinylidene Fluoride membranes. To block the membranes, 5% non‐fat milk in Tris Buffered Saline with Tween‐20 was used, followed by an overnight incubation with primary antibodies and probing with Horseradish Peroxidase‐conjugated secondary antibodies. Signals were detected using a Tanon ECL system. Primary antibodies: PADI4 (17373‐1‐AP, Proteintech), PRMT2 (A5835, Abclonal), ID1 (YP‐Ab‐04970, UpingBio), ID2 (YP‐Ab‐07311, UpingBio), ID3 (YP‐Ab‐07306, UpingBio), H3R8me2a (A3157, Abclonal), GAPDH (A19056, Abclonal), β‐actin (AC026, Abclonal), Citrulline (SMC‐5018, Stressmarq Biosciences), Flag (AE169PM, Abclonal), HA (AE105, Abclonal), CD133 (A0219, Abclonal), Nanog (101287‐T32, Sinobiological), His (AE086, Abclonal), USP7 (A3448, Abclonal), IgG (AC005, Abclonal) and CD44 (60224‐1‐Ig, Proteintech).

### 2.8 Co‐immunoprecipitation assay

The protein lysate extracted was precleared with protein A/G plus agarose (sc‐2003, Santa Cruz) mixed with either a specific primary antibody or control immunoglobulin IgG (AC011, Abclonal) on a rotating device at 4°C overnight. After being washed four times with ice‐cold lysis buffer, the immunoprecipitates were subjected to SDS‐PAGE and analysed via western blotting.

### 2.9 Immunohistochemistry

Sections of tissue were deparaffinized, rehydrated, and exposed to hydrogen peroxide (3%) for 20 min to suppress endogenous peroxidase activity. Sections were first heated in a 0.01 M citrate buffer (pH 6.0) for 15 min to retrieve antigens. Subsequently, they were blocked using 10% goat serum in PBS.

### 2.10 Immunofluorescence staining

For immunofluorescence, the Flag‐PADI4 plasmid was transfected into ECA109 cells, and the cells were harvested for staining after 48 h. Cells were cultured in 12‐well plates. Then cells were fixed in 4% paraformaldehyde (30 min). Subsequently, 0.1% Triton X‐100 was used for 10 min and blocked with 5% Bovine Serum Albumin for 1 h. Then incubated with primary antibodies overnight at 4°C, followed by secondary antibodies for fluorescence imaging. Nuclei were stained with 4′,6‐Diamidino‐2‐phenylindole (DAPI), and images were captured using a Carl Zeiss LSM 700 confocal microscope.

### 2.11 Transwell assay

About 30 000 cells were introduced into the upper chamber, which had been pre‐coated with a Matrigel filter. The medium in the lower chamber was composed of 20% FBS. After incubating for 24 h, it was then fixed using 4% paraformaldehyde. Using a light microscope, the cells stained with crystal violet were visualized and quantified.

### 2.12 Clonogenic assay

Between 1000 and 5000 cells were placed in 6‐well plates and then incubated at 37°C for 10 to 14 days. Following treatment, the cells were washed twice with a complete medium and then incubated at 37°C for 10–14 days. Colonies exceeding 50 cells were considered viable and were quantified using an inverted microscope after crystal violet staining.

### 2.13 In vitro dilution assay

Separation of CD133 positive OSCC cells with magnetic beads. CSCs were seeded into 96‐well plates at various cell densities, including 10, 20, 30, 40 and 50 cells per well, with repeated 10 times. After 14 days, neurosphere formation was assessed, and the sphere‐forming efficiency was calculated using the Extreme Limiting Dilution Analysis software (http://bioinf.wehi.edu.au/software/elda).

### 2.14 In vitro citrullination assay

A total of 1000 ng of recombinant PADI4 (HY‐P70990, MedChemExpress Technology) was incubated with either Flag‐PRMT2 wild‐type or mutant in a 200 µL buffer containing 5 mM DTT, 10 mM CaCl_2_, and 50 mM NaCl at 37°C for 4 h. The presence of protein‐bound citrulline was subsequently detected using a modified citrulline antibody, specifically the Anti‐Citrulline antibody (StressMarq Biosciences, SMC‐501D).

### 2.15 Mass spectrometry

Mass spectrometry was analysed at Jikai company located in Shanghai, China. In a manner similar to the co‐immunoprecipitation assay, HEK 293T cell protein extracts were exposed to anti‐PADI4 and anti‐PRMT2 antibodies, followed by protein A/G agarose bead treatment. Proteins related to PADI4, PRMT2 or IgG were separated through agarose gel electrophoresis.

### 2.16 Tumourigenesis and lung metastasis in nude mice

BALB/c nude mice (6‐week‐old) were used with the approvement from the Tongji University Animal Care and Use Committee (approval no. TJBB05222101). Measurements of tumours were conducted every three days with digital calipers, and volume = (width)^2^ × length × 0.5. Following AVMA Guidelines, euthanasia was carried out using an intraperitoneal injection with a triple dose of barbiturates. Later, the tumours were measured for weight. The tumour was then halved into two approximately equal pieces. One part was preserved in 10% formaldehyde for later IHC staining, while the other part was set aside for western blot. To assess the in vivo impact of the inhibitors GSK484 and cisplatin on OSCC, GSK484 was given 4 mg/kg/2 days for 14 days, while cisplatin was administered at 3 mg/kg on the same schedule. The mice were euthanized 30 days post‐injection, and their tumours were removed. The tumour samples were then fixed in 10% formaldehyde and prepared for haematoxylin and eosin (HE) staining. The lung metastasis test involved BALB/c nude mice. Every mice was injected into 1 million cells in 100 µL of PBS via tail vein. Three weeks after injection, endpoint measurements were taken unless severe illness required earlier euthanasia. The lungs were harvested, washed with saline and fixed in paraformaldehyde. Using a stereoscopic microscope, the number of pulmonary tumour nodules was assessed.

### 2.17 Statistical analysis

Mean ± standard error (SE) were used to present the data. We used Student's *t*‐test for comparing pairs and one‐way analysis of variance for analysing several groups. Correlation analysis was performed using Spearman's or Pearson's methods. Differences from the control group are significant and marked by * for *p* < .05, ** for *p* < .01 and *** for *p* < .001.

## 3 RESULTS

### 3.1 Highly expressed of PADI4 in OSCC and promotes its progress

**FIGURE 1:**
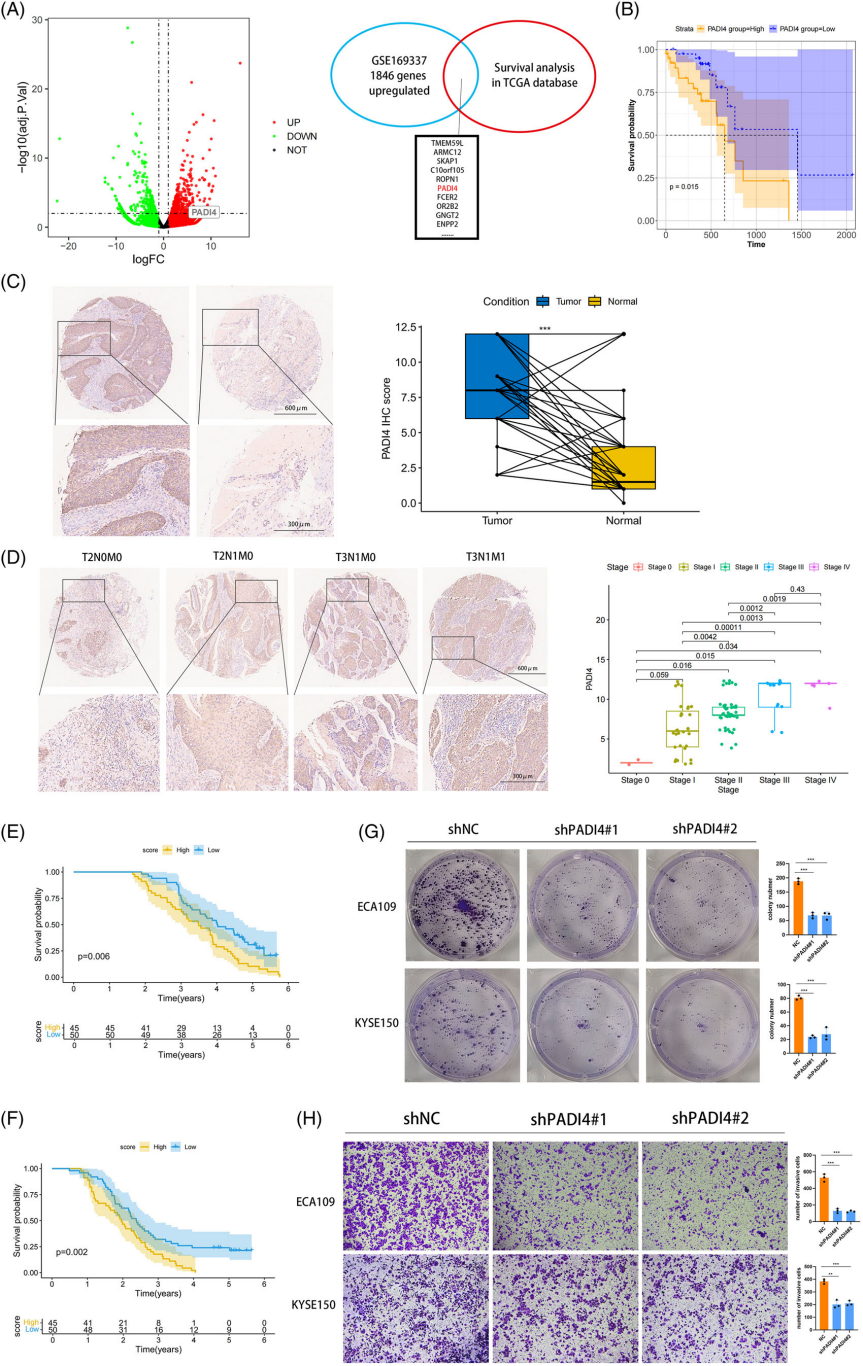
Peptidylarginine deiminase 4 (PADI4) is highly expressed in oesophageal squamous cell carcinoma (OSCC) tissues and linked to tumour stage and poor prognosis. (A) Volcano plot showing gene expression differences between cisplatin‐resistant and parental groups in GSE169337. (B) Kaplan–Meier survival analysis of OSCC patients with different PADI4 levels using The Cancer Genome Atlas (TCGA) data. (C) Immunohistochemical (IHC) analysis comparing PADI4 expression in OSCC tissues to normal oesophageal tissues. (D) IHC analysis of PADI4 expression across TNM stages in OSCC. (E, F) Kaplan–Meier analysis of overall and recurrence‐free survival in OSCC patients with varying PADI4 expression from IHC data. (G) PADI4 knockdown markedly inhibited the proliferation ability of ECA109 and KYSE150 cells indicated by colony formation assay. (H) PADI4 knockdown markedly inhibited the migration ability of ECA109 and KYSE150 cells indicated by transwell assay.

The OSCC dataset was sourced from the GEO database with accession number GSE169337. This dataset comprised two pairs of cisplatin‐resistant and parental OSCC cell lines. Subsequent to conducting GEO2R analysis, differentially expressed genes were illustrated in a volcano plot. We examined the survival of genes with LogFC > 2 in the GSE169337 dataset from the TCGA OSCC. Genes with *p*‐values below  .05 were identified, and the top 10 genes ranked by LogFC are shown in Figure 1A. Finally, PADI4 was chosen for further analysis. Kaplan–Meier analysis was conducted to evaluate the overall survival of OSCC patients based on PADI4 expression levels, utilizing data from the TCGA database and an OSCC tissue microarray (Figure 1B). Immunohistochemistry assays on the OSCC tissue microarray demonstrated significant PADI4 protein overexpression in OSCC tissues compared to normal oesophageal epithelial tissues (Figure 1C). Analysis of the correlation between clinicopathologic parameters and PADI4 levels in OSCC tissues revealed elevated PADI4 protein levels in Stages III and IV cancers compared to Stages I and II, indicating a positive association with tumour stage (Figure 1D). Elevated PADI4 expression correlates with poor overall and recurrence‐free survival revealed by Kaplan–Meier analysis, as demonstrated by IHC data of OSCC tissues (Figure 1E,F). The expression of PADI4 is correlated with lymph node metastasis and distant metastasis in patients undergoing IHC analysis in Table 1. Univariate and multivariate regression analysis showed that PADI4 was an independent prognostic factor of OSCC (Table 2). We chose ECA‐109 and KYSE‐150 cell lines, which exhibit high PADI4 expression, for further investigation, see Figure S1A. Significant decrease in tumour cell proliferation after PADI4 knockdown was demonstrated via colony formation assay (Figure 1G). After PADI4 knockdown, transwell experiments revealed a significant decline in the invasive and migratory capacities of ECA‐109 and KYSE‐150 cells (Figure 1H).

**TABLE 1:** Clinical characteristics of patients and expression of peptidylarginine deiminase 4 (PADI4).

	Low (*n* = 52)	High (*n* = 46)	*p*‐value
Age (mean [SD])	60.60 (9.45)	64.46 (7.00)	.025
Gender (%)			
Female	12 (23.1)	8 (17.4)	.656
Male	40 (76.9)	38 (82.6)	
AJCC (%)			
0	2 (3.8)	0 (0.0)	<.001
I	23 (44.2)	8 (17.4)	
II	25 (48.1)	19 (41.3)	
III	2 (3.8)	13 (28.3)	
IV	0 (0.0)	6 (13.0)	
T (%)			
Tis	2 (3.8)	0 (0.0)	.1
T1	6 (11.5)	2 (4.3)	
T2	18 (34.6)	10 (21.7)	
T3	26 (50.0)	33 (71.7)	
T4	0 (0.0)	1 (2.2)	
N (%)			
N0	50 (96.2)	25 (54.3)	<.001
N1	2 (3.8)	21 (45.7)	
M (%)			
M0	52 (100.0)	40 (87.0)	.023
M1	0 (0.0)	6 (13.0)	

Abbreviation: AJCC, American Joint Committee on Cancer.

**TABLE 2:** Regression analysis of oesophageal squamous cell carcinoma (OSCC) patients.

	Unicox regression analysis			Multicox regression analysis		
	HR	95% CI	*p*‐value	HR	95% CI	*p*‐value
PADI4	1.158	1.071–1.251	<.01	1.165	1.046–1.298	.006
Age	1.035	1.007–1.063	.013			
Gender	1.499	0.864–2.602	.15			
AJCC	1.418	1.038–1.936	.028			
T	1.267	0.877–1.83	.208			
N	1.318	0.79–2.198	.29			
M	8.038	3.263–19.796	<.01	9.013	1.614–50.338	.012
Positive lymph node number	1.069	0.735–1.557	.726			
Tumour volume	1.008	0.995–1.021	.226			
Diameter of tumour	1.068	0.933–1.224	.339			

Abbreviations: AJCC, American Joint Committee on Cancer; CI, confidence interval; HR, hazard ratio; PADI4, peptidylarginine deiminase 4.

### 3.2 PADI4 induces CSC‐like phenotypes and cisplatin resistance in OSCC

**FIGURE 2:**
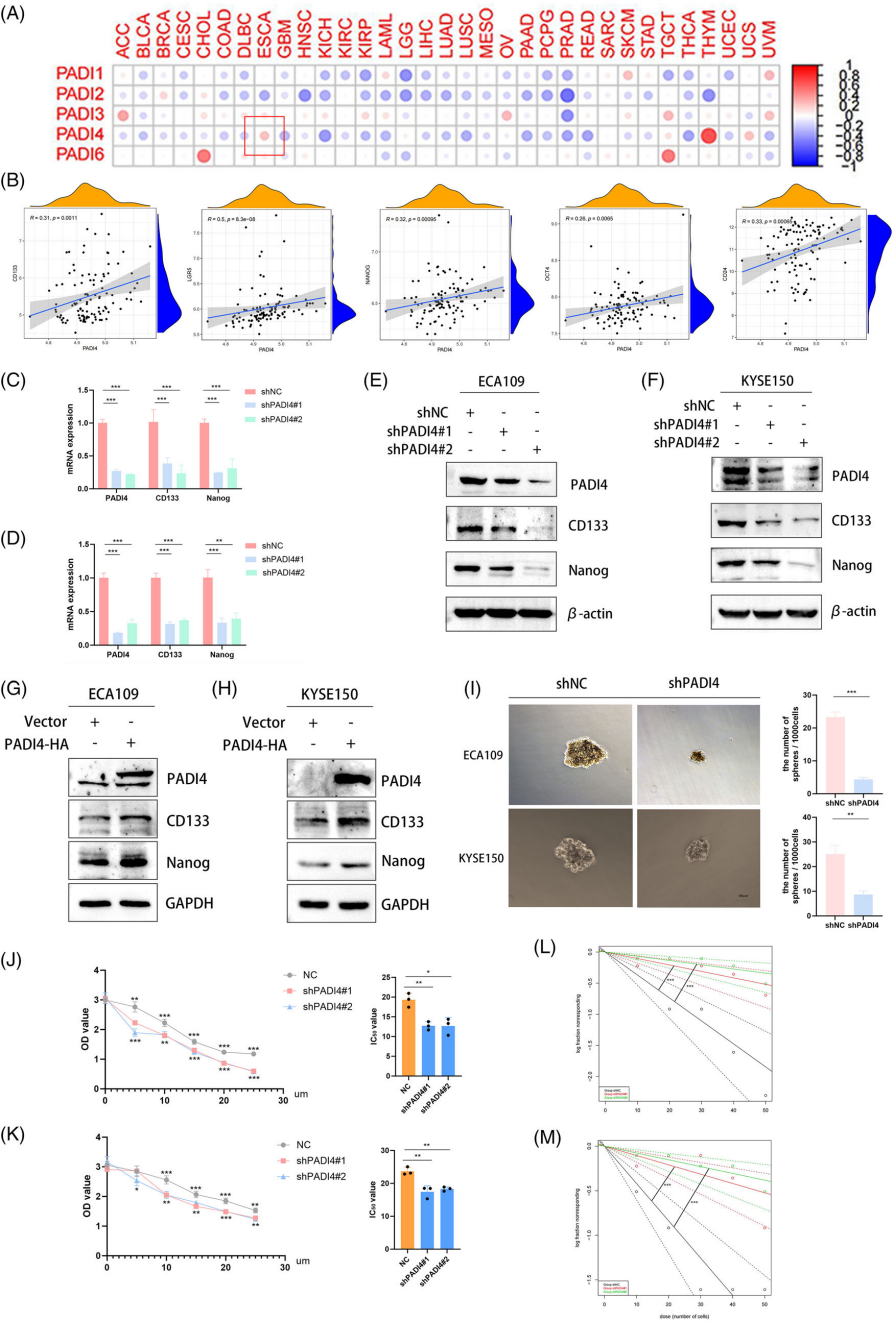
Cancer stem cell (CSC)‐like phenotypes and cisplatin resistance of oesophageal squamous cell carcinoma (OSCC) cells were suppressed by peptidylarginine deiminase 4 (PADI4) knockdown. (A) Comprehensive analysis of PADIs and the stemness across 33 tumour types within the The Cancer Genome Atlas (TCGA) database. (B) Correlation between PADI4 and stemness markers (CD133, LGR5, Nanog, OCT4 and CD24) in GSE23400. (C, D) qPCR showed PADI4 knockdown significantly reduced CD133 and Nanog mRNA levels in ECA109 (C) and KYSE150 (D) cells. (E, F) Western blot revealed PADI4 knockdown significantly decreased CD133 and Nanog protein levels in ECA109 (E) and KYSE150 (F) cells. (G, H) Western blot showed PADI4 plasmids significantly increased CD133 and Nanog protein levels in ECA109 (G) and KYSE150 (H) cells. (I) PADI4 knockdown significantly reduced sphere formation in ECA109 and KYSE150 cells. (J, K) Downregulation of PADI4 enhanced the resistance of ECA109 (J) and KYSE150 (K) cells to cisplatin through CCK8 assay. (L, M) Limiting dilution assays showing the self‐renewing capacity of PADI4 downregulated in CD133‐positive cells of ECA109 and KYSE150.

Through a comprehensive analysis of PADIs and the stemness across 33 tumour types within the TCGA database, it has been determined that PADI4 exhibits a significant association with the stemness characteristics of OSCC (Figure 2A). In addition, an investigation into the correlation between PADI4 and stemness markers, including CD133, LGR5, Nanog, OCT4 and CD24, was conducted using the dataset of GSE23400. The results showed the positive correlation between PADI4 and the stemness markers (Figure 2B). We created and confirmed the effectiveness of shRNA for PADI4 knockout in ECA109 and KYSE150 cell lines. qPCR (Figure 2C,D) and western blotting (Figure 2E,F) analyses showed that PADI4‐KO cells had lower expression levels of stem cell markers CD133 and Nanog compared to control cells. Overexpressing PADI4 led to elevated expression of stem cell markers in ECA109 and KYSE150 cell lines (Figure 2G,H and Figure S1B,C). PADI4‐KO cell lines were observed reducing the ability of sphere formation indicative of the CSCs population (Figure 2I). Furthermore, PADI4 knockout demonstrated enhanced chemoresistance to cisplatin in ECA109 and KYSE‐150 cells. PADI4 knock‐down decreased the IC_50_ of OSCC cells to cisplatin, suggesting that reducing PADI4 expression increases the sensitivity of OSCC cells to cisplatin (Figure 2J,K). We isolated CD133‐positive oesophageal CSCs using magnetic beads to evaluate PADI4's effect on their self‐renewal capacity. The self‐renewal potential of these CD133‐positive oesophageal CSCs was evaluated through an in vitro limiting dilution assay. Figure 2L,M illustrates that the PADI4 knockout group exhibited a significantly reduced cell self‐renewal ability compared to the control group.

### 3.3 PADI4 interacts with PRMT2 and maintains stability of PRMT2

**FIGURE 3:**
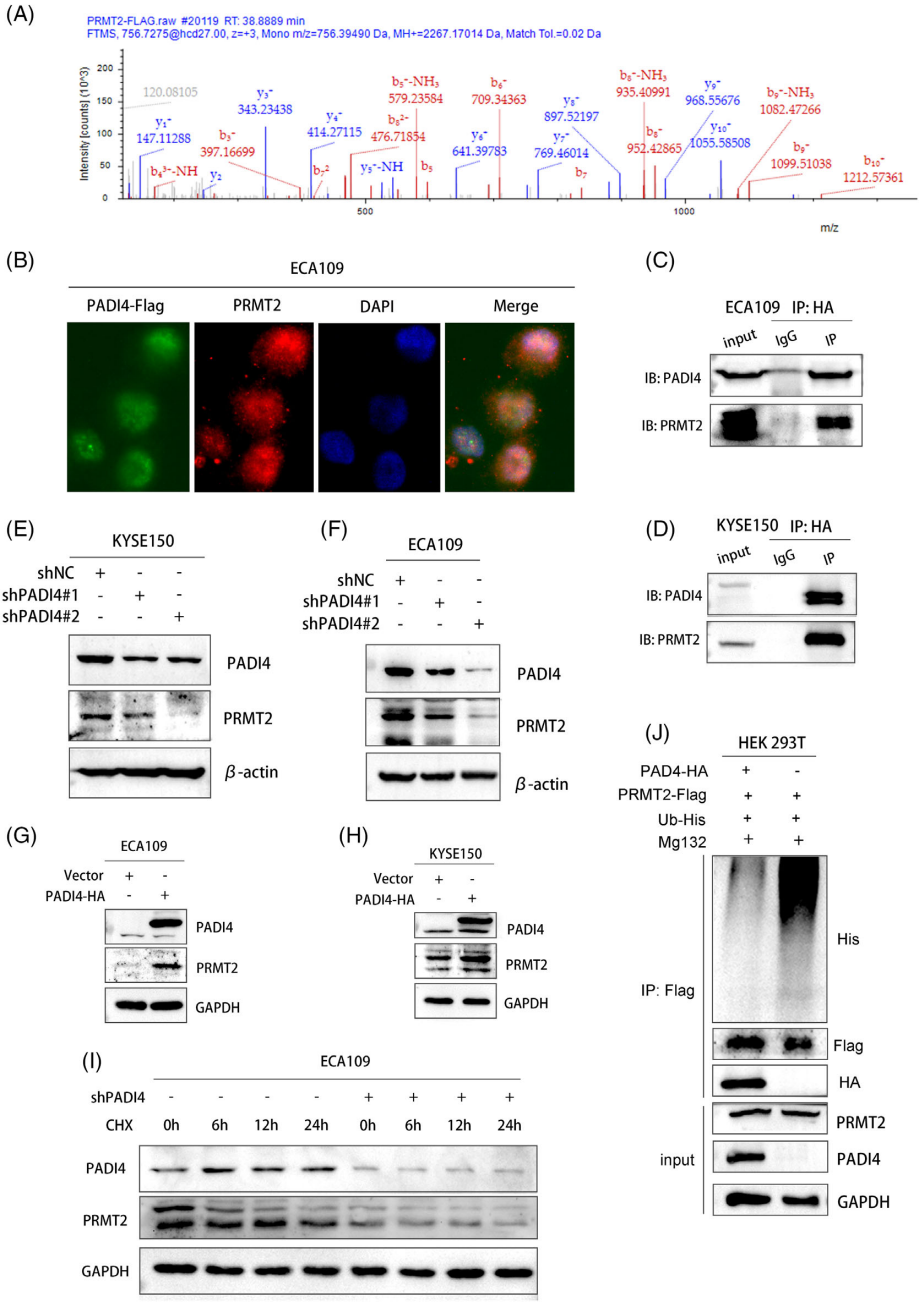
Peptidylarginine deiminase 4 (PADI4) interacts with protein arginine methyltransferase 2 (PRMT2) and maintains stability of PRMT2. (A) Mass spectrometry analysis identified PRMT2 bound with PADI4. (B) Immunostaining of ECA109 cells transfected with PADI4‐HA and PRMT2‐Flag was performed using anti‐HA and anti‐Flag antibodies, with confocal microscopy revealing the subcellular localization of PADI4‐Flag (green), PRMT2 (red) and DAPI (blue, nucleus marker). (C, D) Endogenous cimmunoprecipitation was used to study the interaction between PADI4 and PRMT2 in ECA109 and KYSE‐150 cells after transformed into plasmid of PADI4‐HA. (E, F) PRMT2 protein level in PADI4‐knockdown cell lines. (G, H) PRMT2 protein level in PADI4‐upregulated cell lines. (I) PRMT2 protein degradation levels were measured in treat with cycloheximide (CHX) (10 µg/mL) over specified time intervals in PADI4‐KO ECA109 cells. (J) Lysates from cells co‐transfected with His‐Ub, Flag‐PRMT2 and PADI4‐HA underwent immunoprecipitation with a Flag antibody, followed by immunoblotting with an anti‐His antibody in HEK 293T cells. DAPI, 4′,6‐Diamidino‐2‐phenylindole.

To elucidate PADI4's biological functions and its involvement in OSCC development, PADI4‐HA was overexpressed in cells. The PADI4‐associated protein complex was isolated using tandem affinity purification followed by mass spectrometry analysis. Further, we combined the molecule binding to PADI4 and the citrullinated site analysis (Figure S1D). Mass spectrometry analysis showed that PRMT2 and PADI4 were bound (Figure 3A). Immunofluorescence staining demonstrated that PADI4 and PRMT2 primarily colocalize within the nucleus of ECA109 cells (Figure 3B). Consistent with the mass spectrometry analysis, endogenous coimmunoprecipitation assays indicated the interaction between PADI4 and PRMT2 in ECA109 and KYSE‐150 cells (Figure 3C,D). In our study, we test the expression of PRMT2 in cell with stable knockdown and overexpression of PADI4. Our findings indicated that PRMT2 expression levels decreased following PADI4 knockdown (Figure 3E,F), whereas they increased with PADI4 overexpression (Figure 3G,H). However, PADI4 did not affect the mRNA level of PRMT2 (Figure S1E–H). Cycloheximide analysis further revealed that PADI4 knockdown reduced the half‐life of PRMT2 in ECA109 cells (Figure 3I). In addition, in vivo ubiquitination experiments in HEK 293T cells were conducted by co‐expressing HA‐PADI4 and Flag‐PRMT2 with His‐tagged ubiquitin, followed by immunoprecipitation assays (Figure 3J). The experiments showed that overexpressing PADI4 decreased PRMT2 ubiquitination.

### 3.4 PADI4 citrullinates PRMT2 to maintain the stability of PRMT2

**FIGURE 4:**
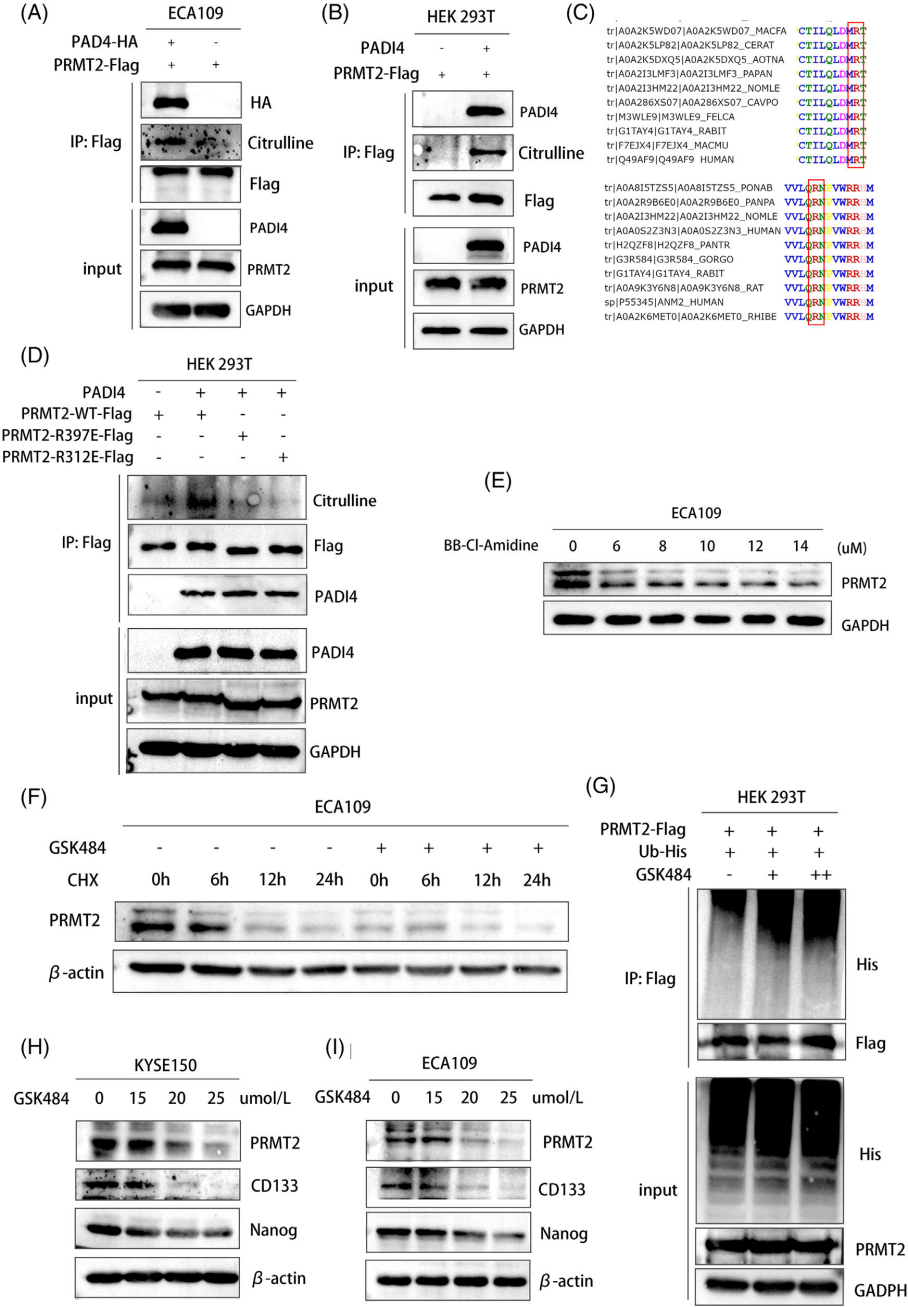
Peptidylarginine deiminase 4 (PADI4) citrullinates protein arginine methyltransferase 2 (PRMT2) to maintain the stability of PRMT2. (A) PADI4‐HA overexpression was immunoprecipitated using Flag‐PRMT2 antibodies, and immunoblotted with modified‐citrulline antibodies. (B) Recombinant PADI4 was immunoprecipitated with a Flag‐PRMT2 antibody in calcium presence, and reaction products were analysed by western blot with modified‐citrulline antibody. (C) The amino acid sequence of PRMT2 in different species is highly conserved at R312 and R397. (D) Two PRMT2 mutant plasmids and transfected into HEK 293T cells to perform immunoprecipitation experiments to investigate citrullination. (E) ECA109 cells were analysed by western blot after 8‐h treatment with varying doses of BB‐Cl‐Amidine. (F) In ECA109 cells treated with GSK484, PRMT2's half‐life was shown by using cycloheximide (CHX) to block protein synthesis. (G) Immunoprecipitating PRMT2 with an anti‐PRMT2‐Flag antibody and probing for polyubiquitination with an anti‐ubiquitin antibody revealed a significant increase in polyubiquitinated PRMT2 protein after GSK484 treatment. (H, I) KYSE150 (H) and ECA109 (I) cells were analysed of cancer stem cell (CSC) markers by western blot after 24‐h treatment with varying doses of GSK484.

Given that PRMT2 is a significant arginine methylase involved in cancer progression, we investigated the hypothesis that PADI4 may directly citrullinate PRMT2. To test this, overexpressing PADI4‐Flag were immunoprecipitated with antibodies against Flag‐PRMT2. Complexes were then immunoblotted using modified citrulline antibody (Figure 4A). We conducted in vitro citrullination assays by incubating recombinant PADI4 with a purified substrate protein transfected with PRMT2. Citrullination was subsequently evaluated using western blotting with an antibody specific to modified citrulline (Figure 4B). Mass spectrometry analysis was used to accurately identify the citrullination sites and R312/R397 was investigated. In addition, the amino acid sequence of PRMT2 in different species is highly conserved at R312 and R397 (Figure 4C). Specifically, we engineered two PRMT2 mutant plasmids and transfected it into HEK 293T cells to perform co‐immunoprecipitation experiments aimed at investigating the interaction with the recombinant PADI4 protein. Our observations revealed a marked reduction in the citrullination of PRMT2 following the mutation of the citrullination sites (R312 and R397) (Figure 4D). The results indicate that citrullinated residues are crucial for PRMT2 and PADI4 interaction. Administration of the pan‐citrullination inhibitor BB‐Cl‐Amidine led to a dose‐dependent reduction in PRMT2 levels in ECA109 cells as BB‐Cl‐Amidine concentrations increased (Figure 4E). This observation suggests that the citrullination process is integral to the stabilization of PRMT2, and that inhibition of this post‐translational modification results in diminished PRMT2 protein levels. In KYSE150 cells treated with specific PADI4 inhibitor (GSK484), PRMT2 exhibited a significantly prolonged half‐life, as demonstrated by the application of cycloheximide inhibit protein synthesis (Figure 4F). By using an anti‐Flag antibody for immunoprecipitation of PRMT2‐Flag and then detecting PRMT2 polyubiquitination with an anti‐ubiquitin antibody, we noted a marked rise in polyubiquitinated PRMT2 protein expression after GSK484 treatment, following MG132 administration (Figure 4G). GSK484 was also found to markedly decrease the expression of PRMT2 downstream targets CSC markers in KYSE‐150 and ECA109 cells with different concentrations (Figure 4H,I).

### 3.5 PRMT2 acts as a positive regulator of IDs family

**FIGURE 5:**
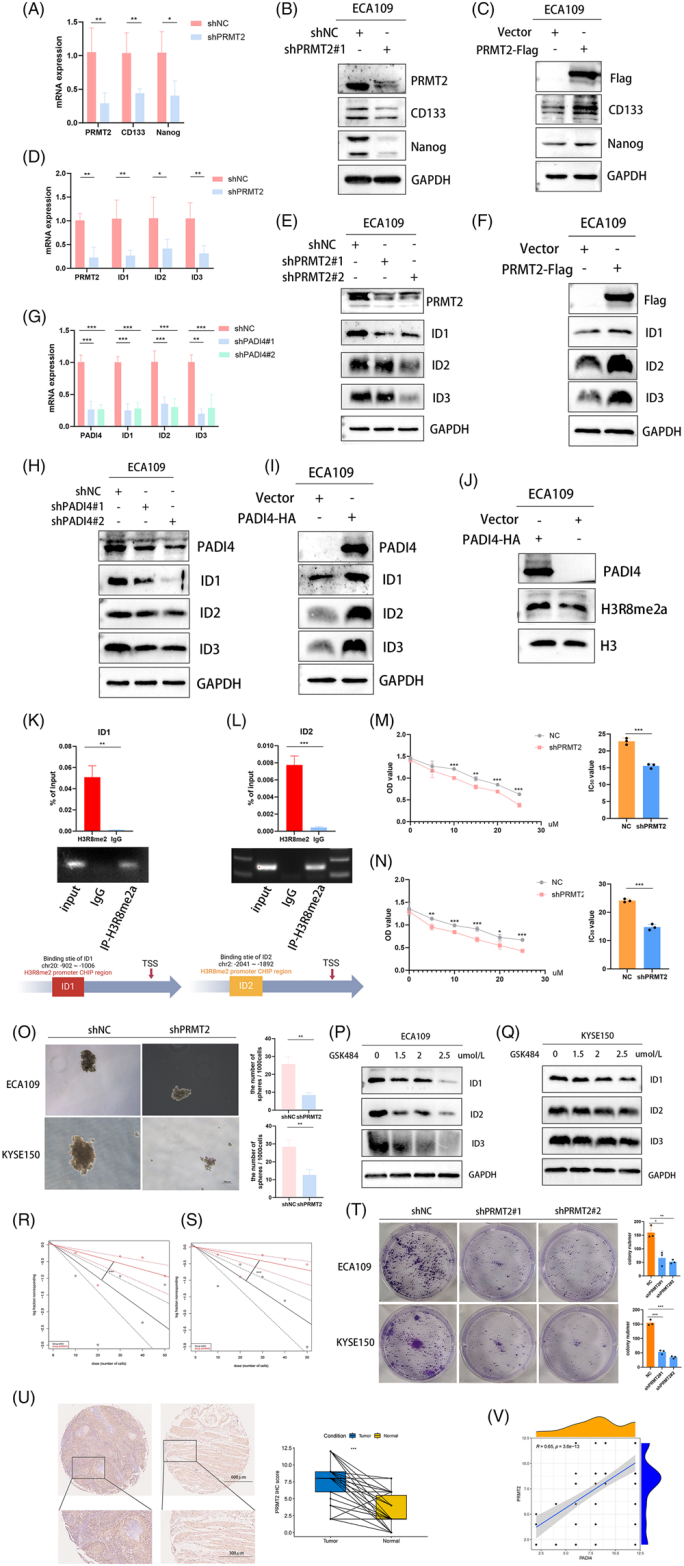
Protein arginine methyltransferase 2 (PRMT2) acts as a positive regulator of inhibitors of DNA binding (IDs). (A, B) The mRNA and protein levels of cancer stem cell (CSC) markers in PRMT2 knockdown ECA109 cells were also assessed by qPCR and western blot. (C) In ECA109 cells transfected with PRMT2 plasmids, the protein levels of CSC markers were measured using western blot. (D, E) The mRNA and protein levels of ID1, ID2 and ID3 in PRMT2 knockdown ECA109 cells were assessed using qPCR and western blot. (F) In ECA109 cells transfected with PRMT2 plasmids, the protein levels of ID1, ID2 and ID3 were measured using western blot. (G, H) The mRNA and protein levels of ID1, ID2 and ID3 in peptidylarginine deiminase 4 (PADI4) knockdown and ECA109 cells were assessed using real‐time PCR and western blot. (I) The protein levels of ID1, ID2 and ID3 in ECA109 cells transfected with PADI4 plasmids were measured. (J) H3R8me2a expression was analysed via western blot in PADI4‐transfected ECA109 cells. (K, L) ChIP assay revealed H3R8me2a enrichment at the human ID1 and ID2 promoter in ECA109 cells. (M, N) Cell viability was assessed using a CCK8 assay after 48‐h treatment with cisplatin in ECA109 (M) and KYSE150 cells (N). (O) PRMT2‐KO cells showed reduced sphere formation in ECA109 and KYSE150 cells. (P, Q) GSK484 reduction in the expression levels of ID1, ID2 and ID3 with different concentration in ECA109 and KYSE150 cells. (R, S) Limiting dilution assays showing the self‐renewing capacity of PRMT2 downregulated in ECA109 (R) and KYSE150 (S) CD133‐positive cells. (T) PRMT2 knockdown markedly inhibited the proliferation ability of ECA109 and KYSE150 cells indicated by colony formation assay. (U) Immunihistochemical (IHC) analysis comparing PRMT2 expression in oesophageal squamous cell carcinoma (OSCC) patients tumour tissues to adjacent normal oesophageal tissues. (V) Correlation between PADI4 and PRMT2 in OSCC patients tumour tissues.

Given the phenotypic changes in shPADI4 cells, we aimed to identify PADI4 targets potentially linked to the development of the CSC phenotype. Analysis of the GSE23400 dataset reveals a positive correlation between PADI4 and stemness markers, such as CD133 and Nanog, with PRMT2 expression (Figure S1I). Our study investigated CD133 and Nanog stem cell marker expression in OSCC cell lines with stable PRMT2 knockdown and overexpression. Our findings indicated that stem cell markers expression levels decreased following PRMT2 knockdown, whereas they increased with PRMT2 overexpression by qPCR (Figure 5A and Figure S1J) and western blot (Figure 5B,C). Subsequently, we used the GEO database (GSE110424) to predict downstream target genes of histone arginine methylation by PRMT2. Stemness‐related transcription factor IDs family (ID1, ID2 and ID3) may be the downstream molecule of PRMT2. Our experiments demonstrated that PRMT2 knockdown significantly reduced mRNA and protein levels of the IDs family (Figures 5D,E), while its overexpression had the opposite effect (Figure 5F and Figure S1K). Similarly, PADI4 knockdown led to a marked decrease in these levels (Figure 5G,H), whereas PADI4 overexpression increased them (Figure 5I, Figure S1L). In addition, western blotting showed that PADI4 overexpression elevated H3R8me2a expression in ECA109 cells (Figure 5J). In order to test whether PRMT2 directly regulates ID1, ID2 and ID3 expression, we analysed the enrichment of PRMT2‐mediated H3R8me2a on the promoters of these candidate genes in ECA109 cells using chromatin immunoprecipitation (ChIP) assays. Notably, ChIP assays provided evidence for the binding of PRMT2 at the ID1 and ID2 promoter region, where the putative PRMT2 response elements are situated. Figure 5K,L illustrates that the core DNA sequence primarily recognized by H3R8me2a is located in the promoter region of the ID1 and ID2 genes. The PRMT2‐KO cells showed increased resistance to cisplatin chemotherapy (Figure 5M,N). In addition, the PRMT2‐KO cell lines exhibited a diminished capacity for sphere formation, which is indicative of a reduction in the CSCs population (Figure 5O). A dose‐dependent decrease in ID1, ID2, and ID3 expression levels was noted with higher GSK484 concentrations (Figure 5P,Q). Taken together, these findings suggest that PRMT2 serves as a positive regulator of IDs family expression, stemness and cisplatin chemotherapy in OSCC. Figure 5R,S illustrates a significant reduction in cell self‐renewal ability in the PRMT2 knockout group compared to the control group. The colony formation assay (Figure 5T) demonstrated a significant decrease in tumour cell proliferation after PRMT2 knockdown. We additionally analysed the expression of PRMT2 in OSCC patient tissues and observed a significant upregulation of PRMT2 in these cancerous tissues (Figure 5U). A notable correlation was observed between PADI4 and PRMT2 expression levels in OSCC (Figure 5V).

### 3.6 USP7 induces stability of PRMT2 expression and spheroid formation of CSC

**FIGURE 6:**
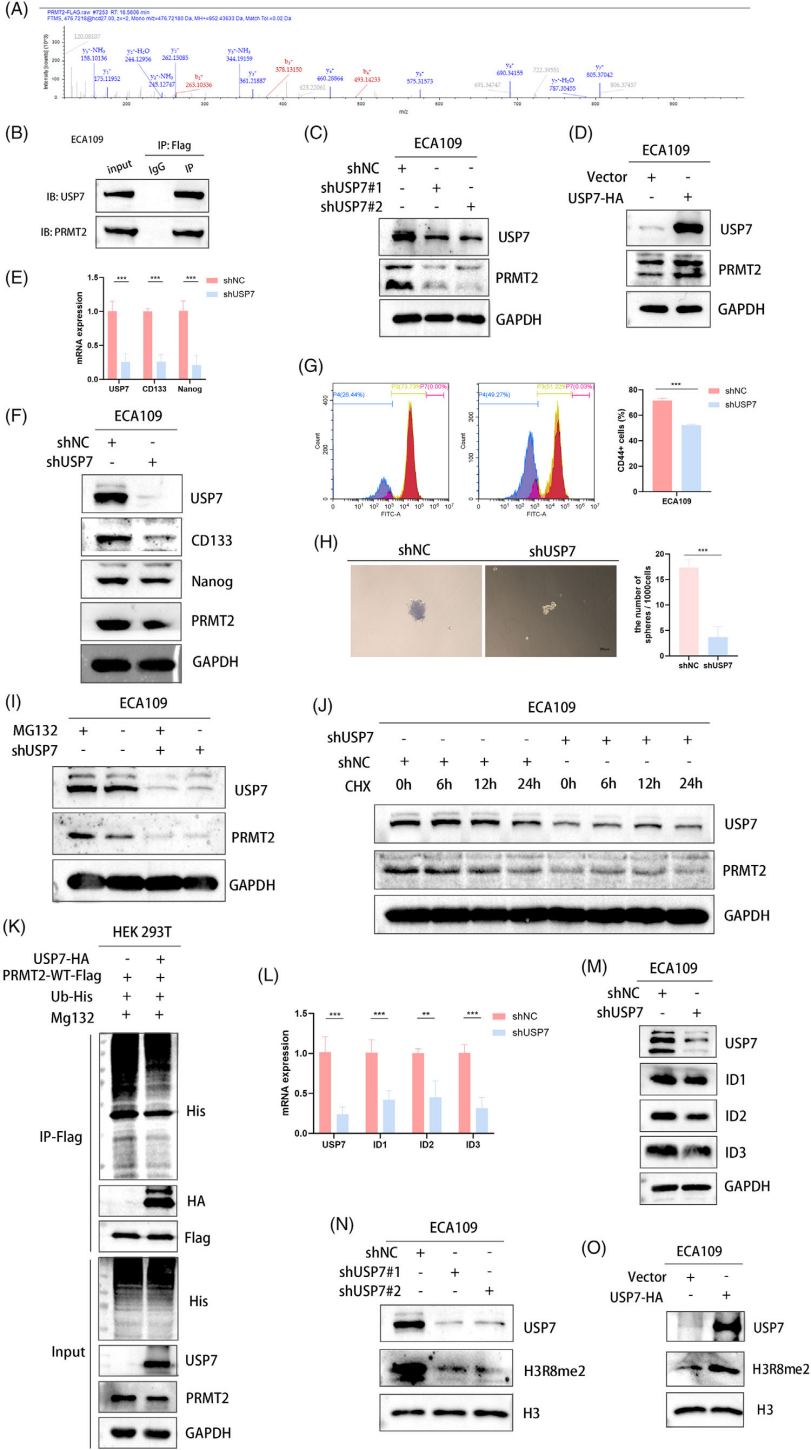
USP7 induces stability of protein arginine methyltransferase 2 (PRMT2) expression and spheroid formation of cancer stem cell (CSC). (A) Mass spectrometry analysis identified PRMT2 as a prospective target of USP7. (B) Conducted endogenous immunoprecipitation to study USP7 and PRMT2 interaction in ECA109 cells after transformed into plasmid of PRMT2‐Flag. (C) USP7‐KO decreased the expression of PRMT2 in ECA109 cell line via western blot. (D) PRMT2 expression was analysed via western blot in ECA109 cells transfected with USP7‐HA. (E) qPCR showed USP7 knockdown significantly reduced Nanog and CD133 and mRNA levels in ECA109 cells. (F) Western blot analysis showed CSC markers decreased in USP7‐KO ECA109 cell. (G) FACS analysis revealed that USP7 knockdown significantly reduced CD44+ cell percentages in KYSE150 cells. (H) USP7 knockdown significantly impaired sphere formation in KYSE150 cells. (I) Western blots of USP7 knockout ECA109 cell treated with DMSO or MG132 (20 µM) for 5 h. (J) ECA109 cells with USP7 knockout were treated with cycloheximide (CHX) (10 µg/mL) for varying durations, and PRMT2 protein levels were assessed by western blotting. (K) HEK 293T cells co‐transfected with HA‐USP7, His‐Ub and PRMT2‐Flag were treated with MG132 (20 µM) for 5 h before harvesting to evaluate PRMT2 ubiquitination. (L, M) qPCR and western blot analysis of IDs from USP7 knockout ECA109 cells. (N) Analyse USP7 knockout cells expressing H3R8me2a. (O) Conduct western blot analysis on lysates with HA‐USP7 to assess H3R8me2a.

To investigate the precise mechanism by which PADI4 deubiquitinates PRMT2, we performed an analysis of potential deubiquitinating enzymes acting upstream of PRMT2. By integrating mass spectrometry data for PADI4 and PRMT2, we identified USP7 as a candidate enzyme potentially interacting with both PADI4 and PRMT2 (Figure 6A). We speculated that citrullination may modulate the interaction between PRMT2 and USP7, thereby impacting the deubiquitination process. Furthermore, the expression levels of PRMT2 are markedly affected by either the overexpression or knockout of USP7. Our investigation further revealed that USP7 is a pivotal molecule influencing stemness. Endogenous co‐immunoprecipitation in ECA109 cells confirmed the interaction between USP7 and PRMT2 (Figure 6B). USP7 knockdown significantly decreased PRMT2 protein levels, as shown in Figure 6C. Conversely, the overexpression of USP7 resulted in an increase in PRMT2 level, as illustrated in Figure 6D. USP7 knockout leads to a marked decrease in the expression of stemness‐related molecules CD133 and Nanog, along with a significant reduction in tumour sphere formation (Figure 6E,F). Flow cytometry analysis indicated a marked reduction in the percentage of CD44+ cells in USP7‐KO cells (Figure 6G). The results indicate that USP7 might influence cancer cell stemness by potentially altering the expression of stemness markers like CD44, CD133, and Nanog, known for their link to the stem‐like traits of cancer cells. Considering that the degradation of PRMT2 is dependent on proteasomal activity, we incorporated the proteasome inhibitor MG132 in our experimental procedures. Our study reveals that USP7 depletion leads to reduced PRMT2 protein levels, an effect mitigated by MG132 treatment (Figure 6I). Moreover, a protein half‐life assay demonstrated that the reduction of USP7 compromised the stability of PRMT2 protein in OSCC cells, as illustrated in Figure 6J (KYSE150 cell line) and Figure S1M (ECA109 cell line). Importantly, a co‐immunoprecipitation revealed that PRMT2 ubiquitination levels were diminished following USP7 overexpressed (Figure 6K). We further investigated the influence of USP7 on its downstream target. USP7 knockdown significantly reduced ID1, ID2 and ID3 expression at both protein and mRNA levels (Figure 6L,M). USP7‐KO significantly decreased the protein level of H3R8me2a, whereas USP7 overexpression produced the opposite effect (Figure 6N,O).

### 3.7 PRMT2 citrullinate disrupts the interaction between USP7 and PRMT2

**FIGURE 7:**
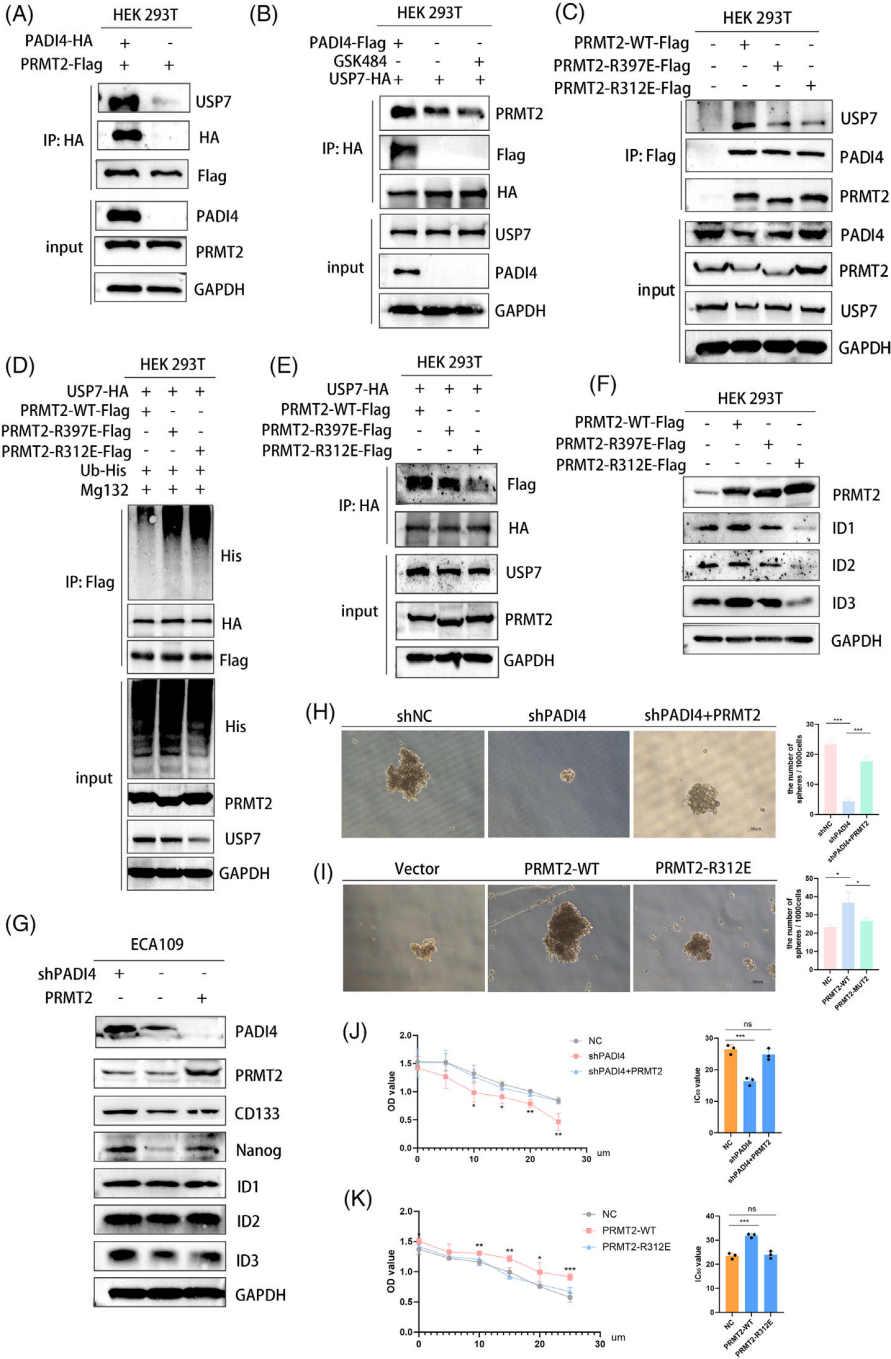
Protein arginine methyltransferase 2 (PRMT2) citrullinate disrupts the interaction between USP7 and PRMT2. (A) HEK 293T cells were co‐transfected with PRMT2‐Flag and PADI4‐HA plasmids, and USP7 levels were assessed via western blot. (B) Co‐transfect HEK 293T cells with PADI4‐Flag and USP7‐HA, treat with GSK484 (20 µM) for 24 h, and use western blot to evaluate PRMT2 levels. (C) HEK 293T cells were co‐transfected with PRMT2‐WT‐Flag, PRMT2‐R312E‐Flag and PRMT2‐R397E‐Flag plasmids, and USP7 levels were assessed via western blot. (D) HEK 293T cells were co‐transfected with HA‐USP7, His‐Ub, PRMT2‐Flag, PRMT2‐R397E‐Flag and PRMT2‐R312E‐Flag, then treated with MG132 (20 µM) for 5 h before harvesting to assess PRMT2‐Flag ubiquitination levels. (E) HEK 293T cells were co‐transfected with HA‐USP7, PRMT2‐WT‐Flag, PRMT2‐R397E‐Flag and PRMT2‐R312E‐Flag, and then use western blot to evaluate Flag levels. (F) Western blot was used to analyse ID1, ID2 and ID3 expression in HEK 293T cells transfected with PRMT2‐WT, PRMT2‐R397E and PRMT2‐R312E. (G) ECA109 cell line transfected plasmid overexpressing PRMT2 or PRMT2‐R312E to evaluate resistance of ECA109 cells to cisplatin. (H) Peptidylarginine deiminase 4 (PADI4) knockdown reduced sphere formation in ECA109 cells and PRMT2 restores this effect. (I) ECA109 cell line transfected plasmid overexpressing PRMT2 or PRMT2‐R312E to evaluate the sphere formation. (J) PADI4 knockdown reduced sphere formation in ECA109 cells and PRMT2 restores this effect. (K) Downregulation of PADI4 enhanced the resistance of ECA109 cells to cisplatin through CCK8 assay and PRMT2 restores this effect.

In order to prove that citrullinization can affect the ubiquitination of PRMT2 by affecting the results of PRMT2 and USP7, we organized the following experiments. The interaction between PRMT2 and USP7 was notably disrupted in cells overexpressing PADI4 (Figure 7A). Similarly, treatment with GSK484 in comparison to control cells resulted in the disruption of the PRMT2‐USP7 interaction (Figure 7B). Furthermore, mutating the arginine sites (R312 and R397) of PRMT2 significantly diminished its binding affinity to USP7 (Figure 7C). To investigate the hypothesis that PRMT2 ubiquitination depends on its citrullination by PADI4, Flag‐tagged PRMT2 mutants R312E and R397E were expressed in HEK 293T cells. The R312E and R397E mutants of PRMT2, unlike the wild‐type PRMT2, significantly increased ubiquitination in HEK 293T cells (Figure 7D). HEK 293T cells were co‐transfected with HA‐USP7, PRMT2‐WT‐Flag, PRMT2‐R312E‐Flag and PRMT2‐R397E‐Flag, and then use western blot to evaluate Flag levels. The mutation of PRMT2 weakened the binding with USP7, especially in R312 site (Figure 7E). Western blot analysis of ID1, ID2 and ID3 expression in HEK 293T cells transfected with PRMT2‐WT‐Flag, PRMT2‐R312E‐Flag and PRMT2‐R397E‐Flag revealed that PRMT2 mutations, particularly at the R312 site, influenced the expression of the ID family (Figure 7F). Furthermore, overexpression of PRMT2 can resume the decrease of IDs family and CSC markers caused by PAID4 knock‐down (Figure 7G). The overexpression of PRMT2 has been shown to resume spheroid formation in ECA109 cells, which are otherwise compromised by PADI4 knockdown (Figure 7H). Similarly, PRMT2 overexpression can also reinstate cisplatin resistance in these cells affected by PADI4 knockdown (Figure 7J). Then, we transfected ECA109 cells with PRMT2‐WT or PRMT2‐R312E to investigate the potential impact of the R312 mutation on spheroid formation and cisplatin resistance. The results discovered that this site of mutation leads to changes in both CSC spheroid formation (Figure 7I) and cisplatin resistance (Figure 7K). These findings indicate that citrullination at PRMT2 residue R312 disrupts the interaction between USP7 and PRMT2, ultimately promoting PRMT2 ubiquitination and subsequent change in both CSCs spheroid formation and cisplatin resistance.

### 3.8 PADI4 knockout overcomes chemotherapy resistance in vivo

**FIGURE 8:**
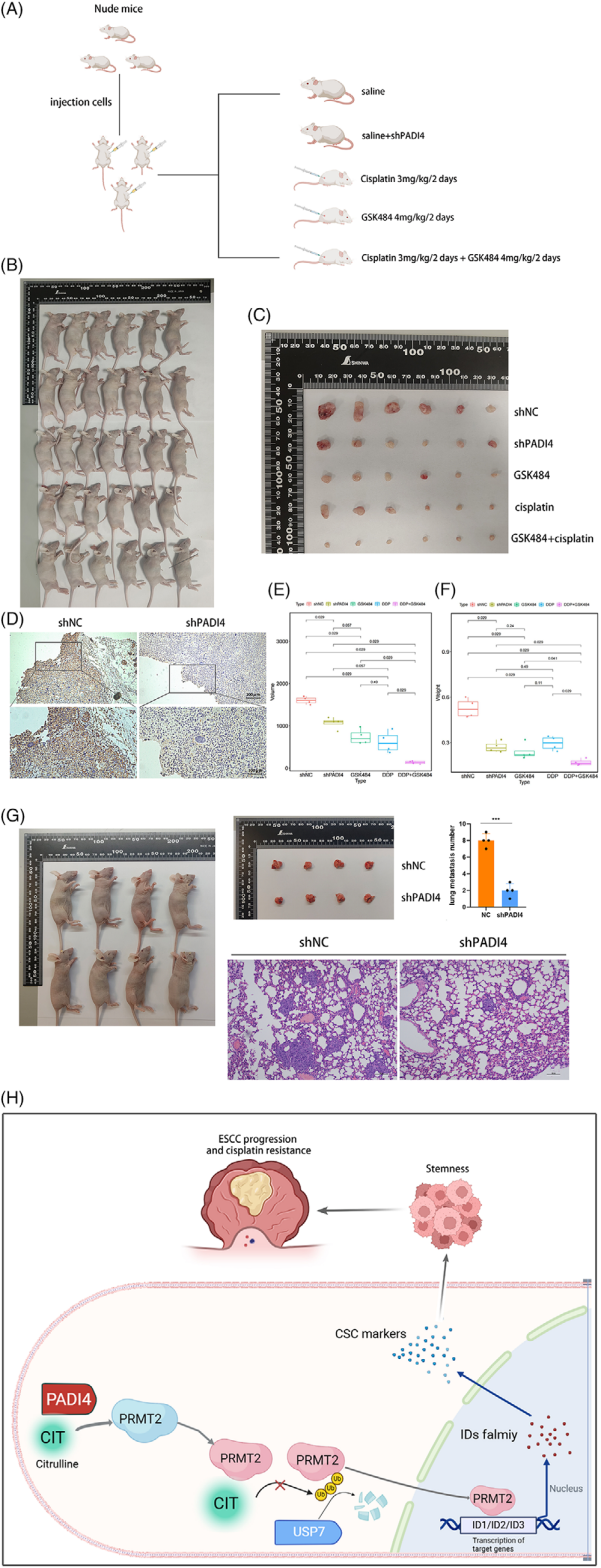
Peptidylarginine deiminase 4 (PADI4) facilitates tumour growth and metastasis in vivo, while GSK484 exhibits a synergistic effect with cisplatin in vivo. (A) A schematic diagram illustrating the treatment protocol for mice in this study is presented. (B, C) Images of excised subcutaneous tumours from mice treated with shPADI4, GSK484 and cisplatin, and the combination of GSK484 and cisplatin are shown. (D) Immunohistochemical (IHC) analysis of PADI4 protein expression in excised tumours from the shPADI4, GSK484, cisplatin and GSK484 plus cisplatin treatment groups is presented. (E) The volume of subcutaneous tumours in the shPADI4, GSK484, cisplatin and GSK484 plus cisplatin treatment groups is depicted. (F) The weight of subcutaneous tumours in the shPADI4, GSK484, cisplatin and GSK484 plus cisplatin treatment groups is reported. (G) HE staining revealed oesophageal squamous cell carcinoma (OSCC) metastases in the lung tissues. (H) The schematic diagram of the underlying mechanism in this study was shown.

In vitro experiments demonstrated that inhibiting PADI4 greatly restored the sensitivity of OSCC cells to cisplatin. Xenograft tumour models were established using KYSE150 cells with either shNC or shPADI4 expression. The shNC group was further divided into treatment subgroups: saline, GSK484, cisplatin and a combination of GSK484 and cisplatin (Figure 8A,B). When the subcutaneous tumour is removed (Figure 8C), IHC staining suggested that shPADI4 group showed silencing of PADI4 population (Figure 8D). Xenograft tumours from shPADI4, GSK484, cisplatin and GSK484 plus cisplatin groups exhibited a significant reduction in both tumour mass (Figure 8E) and weight (Figure 8F) compared to the shNC group. The tumour of GSK484 plus cisplatin is smaller than that of GSK484 or cisplatin alone. The tumour metastasis assay revealed that PADI4 knockdown significantly decreased the average number of lung metastasis nodules, as shown by HE staining (Figure 8G). The findings suggest that removing PADI4 suppresses OSCC growth. The inhibitory effect was significantly amplified when combined with chemotherapy. Figure 8H illustrates the study's underlying mechanism.

## 4 DISCUSSION

Citrullination, particularly facilitated by PADI4 due to its involvement in the formation of neutrophil extracellular traps, is a well‐known modification. However, the current research ignores the important role of citrullination in epigenetics. The involvement of PADI4 in tumour progression has been substantiated by numerous studies.^19^, ^20^, ^21^, ^22^ Based on our prior research on chemotherapy resistance in OSCC and comprehensive database analyses, we curious the potential role for PADI4 in influencing cisplatin resistance and stemness in oesophageal cancer. In this study, utilizing database analysis, we identified a significant correlation between PADI4 expression in OSCC patients and the expression of stemness‐associated molecules, including CD133, Nanog, LGR5, OCT‐4 and CD24. This finding suggests that PADI4 may impact the stemness characteristics of OSCC, potentially contributing to cisplatin resistance. We investigated the impact of PADI4 knockdown on the malignant behaviour of two established OSCC cell lines to evaluate this hypothesis. Our results demonstrated that PADI4 knockdown inhibited the expression of tumour stemness markers (including CD133 and Nanog) and spheroid growth. These findings suggest that PADI4 may influence cisplatin resistance by modulating the tumour stemness of oesophageal cancer cells. Furthermore, this study systematically explores the underlying mechanisms.

A significant discovery of this study is that PADI4 directly citrullinates PRMT2 at the sites of R312 and R397, which affect the ubiquitination of PRMT2 and enhanced its stability. Thereby, PADI4 enhancing PRMT2‐mediated histone methylation modification in OSCC cells. Numerous studies have posited that protein citrullination catalysed by PADI4 is a significant aspect of PADI4's role in cancer pathogenesis.^20^ Historically, research on PADI4 has predominantly concentrated on its function in neutrophils, particularly its role in facilitating neutrophil extracellular trap formation.^23^ Nonetheless, some studies examining the epigenetic modifications mediated by PADIs. Emerging evidence increasingly indicates that citrullination, akin to other epigenetic modifications, has the potential to directly alter downstream proteins. For instance, the citrullination of MEK1 at residues R113/189 by PADI2 represents a pivotal divergence in the context of endothelial cell malignancies.^24^ PADI2 is capable of citrullinating the nuclear antigen Nucleophosmin at position 277, a modification that can be targeted by CD4 T cells for anti‐tumour therapy.^25^ Some of recent studies have acknowledged the influence of PADIs‐mediated citrullination on the ubiquitination and subsequent degradation of downstream molecules. For instance, recent findings indicate that PADI2 plays a protective role in safeguarding the androgen receptor from proteasome‐mediated degradation.^26^ Citrullination is associated with the stability of HIF‐1α, indicating that targeting PADI4‐mediated citrullination of HIF‐1α may represent a promising therapeutic approach for cancers exhibiting aberrant expression of HIF‐1α.^27^ However, current literature indicates that the repertoire of molecules known to be regulated by citrullination remains limited. Our study demonstrated that PADI4 enhances PRMT2 protein expression without affecting its RNA levels. In this novel area of research exploring the impact of citrullination on ubiquitination and protein degradation, our study was the first to demonstrate that the inhibition of citrullination—achieved through GSK484 treatment, PADI4 gene overexpression, or R312/R397 mutation—can influence the de‐ubiquitination of PRMT2. Consequently, our research establishes a connection between citrullination and arginine methylation.

In recent years, the significance of epigenetic modifications, particularly arginine methyltransferases, in tumour biology and chemotherapy sensitivity has become increasingly evident. Notably, PRMT1 is important for the activation of SMAD3 by TGF‐β, much like the methylation of SMAD6 by bone morphogenetic proteins, aiding in TGF‐β‐induced epithelial‐mesenchymal transition and the production of epithelial stem cells.^28^ Furthermore, the PRMT3‐IGF2BP1‐HEG1 axis has been identified as a critical regulator and potential therapeutic target in the context of oxaliplatin resistance.^29^ The PRMT family contains many star molecules, such as PRMT5. Research on PRMT5 is relatively advanced, with studies indicating its significance as a crucial anti‐tumour molecule.^30^ The entire PRMT family is recognized for its important role in tumour biology. In contrast, research on PRMT2, another member of the PRMT family, is still not well understood. Although the regulatory function of PRMT2 in tumourigenesis is fairly established, it is particularly noted for connecting histone H3R8 asymmetric dimethylation to oncogenic activation and glioblastoma development.^18^ However, at present, the research on PRMT2 is still very limited, and there is a lack of high‐quality and in‐depth research. ID family proteins, including ID1, ID2, ID3 and ID4, are influential transcription factors significantly involved in tumourigenesis. Contemporary research on ID family proteins predominantly explores its association with cancer stemness. For instance, downregulation of ID1 expression has been shown to mitigate the progression of colorectal cancer and enhance tumour responsiveness to both immunotherapy and chemotherapy.^31^ The upregulation of DYRK1 results in the phosphorylation of ID2 at threonine 27, subsequently leading to the destabilization of HIF2α. This process contributes to the reduction of glioma stem cell characteristics, suppression of tumour progression, and improved prognosis for patients with glioblastoma.^32^ ID3 enhances intrahepatic cholangiocarcinoma (ICC) stemness by increasing β‐catenin transcriptional activity, indicating its potential as a biomarker for predicting ICC patient responses to adjuvant chemotherapy.^9^ Combined with the current researches, the effect of IDs family on tumour stemness is widely recognized. Database predictions suggest that PRMT2 regulates histone arginine methylation, with ID1, ID2 and ID3 possibly serving as downstream targets. Using qPCR and western blot analyses, we initially identified and validated that alterations in PRMT2 influence the transcription and expression of ID1, ID2 and ID3. Subsequent chromatin immunoprecipitation experiments further substantiated PRMT2's role in the transcriptional regulation of ID1 and ID2. Considering the alterations in PRMT2 that influence the transcription of ID1, ID2, and ID3, we demonstrated that PRMT2 may also regulate the tumour stemness of OSCC. Given the absence of prior reports on the role of PRMT2 in IDs family, our study is first to demonstrate that PRMT2 regulates IDs family transcription, thereby influencing its expression, which showed that PRMT2 may also regulate the tumour stemness of OSCC.

Consequently, the mechanism by which PADI4‐mediated citrullination affects cisplatin resistance might involve modulating the IDs family and tumour stemness of OSCC through the de‐ubiquitination of PRMT2. This study found that inhibiting PRMT2 citrullination or mutating PRMT2 at R312/R397 prevents the upregulation of IDs family expression, indicating that PADI4‐catalyzed citrullination of PRMT2 regulates the IDs family. Furthermore, the application of citrullination inhibitors effectively reduces IDs family expression. Our findings suggest that the IDs family acts as a downstream effector in the PADI4/PRMT2 signalling pathway. Furthermore, we have conducted an in‐depth investigation into the specific mechanisms by which PADI4‐mediated ubiquitination regulates PRMT2. Through the application of mass spectrometry analysis and co‐immunoprecipitation experiments, we have conclusively verified the deubiquitination role of USP7 on PRMT2. Notably, the deubiquitination function of USP7 on PRMT2 has not been previously documented. Previous research on post‐translational modifications has indicated that modifications such as phosphorylation and acetylation influence the ubiquitination and deubiquitination processes by modulating the interaction between substrate molecules and E3 ligases. Based on these results, we propose that the deubiquitination activity of PADI4 on PRMT2 is similarly mediated by its impact on the binding affinity of PRMT2 to E3 ligases. This hypothesis was substantiated through co‐immunoprecipitation and ubiquitination assays, the regulation of PADI4 on PRMT2 occurs through the modulation of USP7's binding to PRMT2, subsequently influencing the deubiquitination activity of USP7 on PRMT2. Further research revealed that the R312 and R397 residues are critical in this regulatory mechanism. Especially, the mutation of R312 site of PRMT2 can significantly affect the sphere formation and cisplatin resistance of OSCC cells. Nevertheless, our study is subject to certain limitations. Firstly, there is an absence of flow cytometry validation for tumour stem cell marker CD133 and other relevant indicators. Secondly, our research does not encompass investigations into drug‐resistant cell lines derived from oesophageal cancer. Lastly, there is a lack of additional clinical datasets to support the prognostic evaluation and clinical validation of PADI4. We hope to further improve it in the future research.

In conclusion, this study elucidates the regulation of PRMT2 by PADI4‐mediated citrullination, identifying specific citrullination sites. It further demonstrates that the IDs protein family acts as a downstream effector modulated by PRMT2 arginine methylation. This study is the first to link protein citrullination with the regulation of arginine methylation, suggesting their potential involvement in cancer cell progression. These insights enhance the comprehension of how PADI4 affects tumour stemness through PRMT2 citrullination. Therefore, our findings open up the possibility of using citrullination inhibitors to regulate tumour stemness to inhibit tumour progression and cisplatin resistance.

## AUTHOR CONTRIBUTIONS

Zeyu Wang, Hao Wu and Zhaoxing Li equally contributed to the experiments. Meidong Xu and Tao Chen designed and revised the study. Anqi Feng, Yuan Chu, Kang Fang and Zehua Zhang handled clinicopathological analysis. Ziying Zhao, Zhuyun Leng, Zhukai Chen, Shihan Zhang and Xiaoyuan Wang conducted bioinformatics analysis. Zeyu Wang and Lingnan He analysed the data and wrote the paper. All authors reviewed and approved the final manuscript.

## CONFLICT OF INTEREST STATEMENT

The authors declare no conflicts of interest.

## ETHICS STATEMENT

This study was performed in line with the principles of the Declaration of Helsinki. Approval was granted by the Ethics Committee of Shanghai East Hospital, School of Medicine, Tongji University.

## Supporting information



Supporting Information

Supporting Information

## Data Availability

The data that support the findings of this study are available from the corresponding author upon reasonable request.
