# Supplementary figures and images for: PADI4 facilitates stem‐like properties and cisplatin resistance through upregulating PRMT2/IDs family in oesophageal squamous cell carcinoma
Source: Clin Transl Med. 2025 Mar 13;15(3):e70272. doi: 10.1002/ctm2.70272 (PMC11904308; doi:10.1002/ctm2.70272)

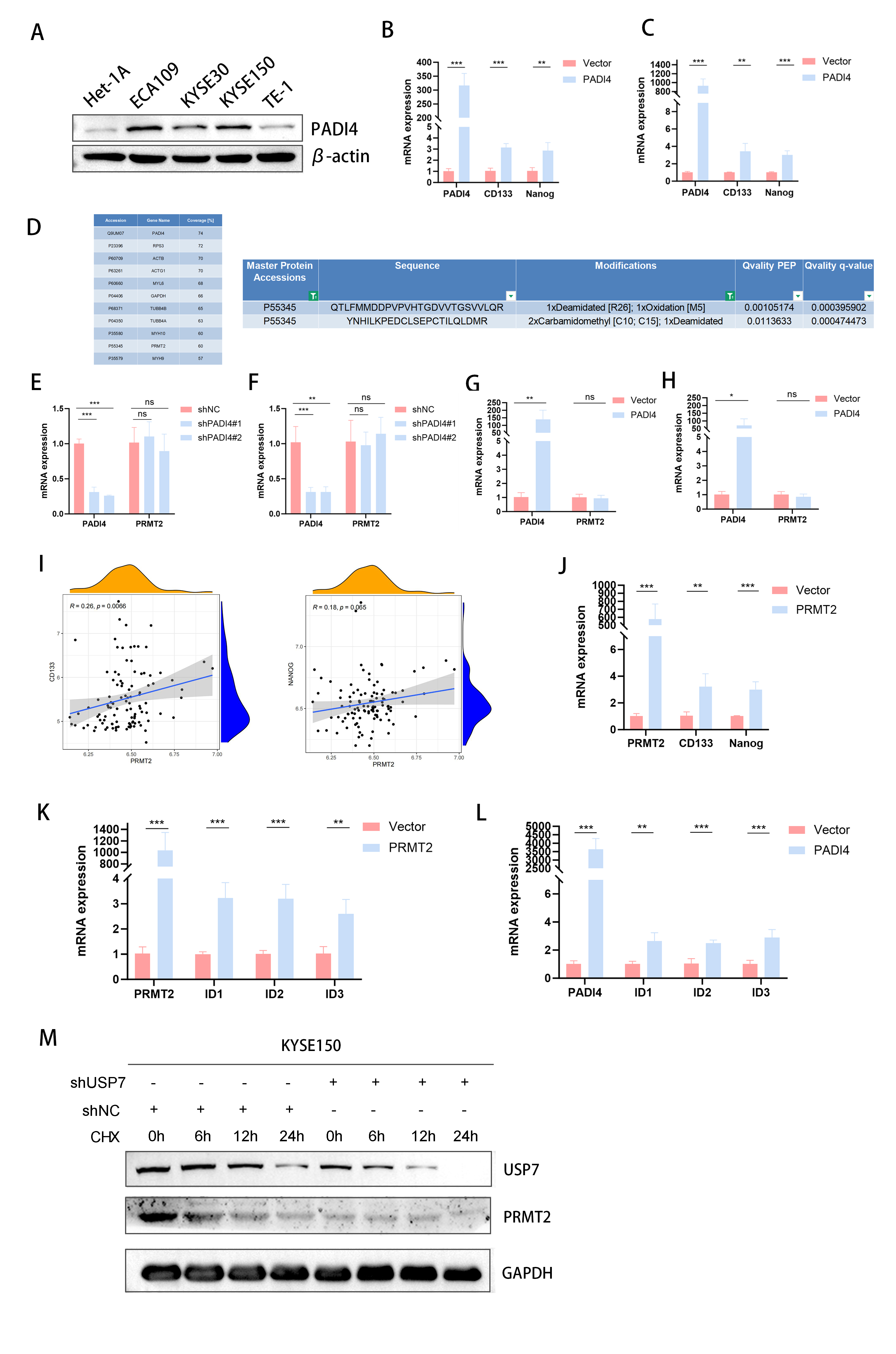

Supplement: Supplementary file 1 — Supporting Information [file CTM2-15-e70272-s002.jpg]
