# Supplementary material for: PADI4 facilitates stem‐like properties and cisplatin resistance through upregulating PRMT2/IDs family in oesophageal squamous cell carcinoma
Source: Clin Transl Med. 2025 Mar 13;15(3):e70272. doi: 10.1002/ctm2.70272 (PMC11904308; doi:10.1002/ctm2.70272)
Supplement: Supplementary file 2 — Supporting Information [file CTM2-15-e70272-s001.docx]

Supplementary Table 1. Sequences of each assay.

| Assay | Name | Sequence |
| --- | --- | --- |
| RT-qPCR | PADI4-F | CAGGGGACATTGATCCGTGTG |
|  | PADI4-R | GGGAGGCGTTGATGCTGAA |
|  | PRMT2-F | ACATTCCGGCAAACCATGTG |
|  | PRMT2-R | GGATGACTTTATCCGTCAGGGA |
|  | ID1-F | CTGCTCTACGACATGAACGG |
|  | ID1-R | GAAGGTCCCTGATGTAGTCGAT |
|  | ID2-F | AGTCCCGTGAGGTCCGTTAG |
|  | ID2-R | AGTCGTTCATGTTGTATAGCAGG |
|  | ID3-F | GAGAGGCACTCAGCTTAGCC |
|  | ID3-R | TCCTTTTGTCGTTGGAGATGAC |
|  | CD133-F | AGTCGGAAACTGGCAGATAGC |
|  | CD133-R | GGTAGTGTTGTACTGGGCCAAT |
|  | Nanog-F | TTTGTGGGCCTGAAGAAAACT |
|  | Nanog-R | AGGGCTGTCCTGAATAAGCAG |
|  | USP7-F | GGAAGCGGGAGATACAGATGA |
|  | USP7-R | AAGGACCGACTCACTCAGTCT |
|  | CD44F | CCAGCCTCTGCCAGGTTC |
|  | CD44R | CCAATAAGTGCTTTCAACTCAGCA |
|  | OCT4-F | CTTGAATCCCGAATGGAAAGGG |
|  | OCT4-R | GTGTATATCCCAGGGTGATCCTC |
| Chromatin immunoprecipitation | ID1-F | GGGAGAACAAGACCGATCGG |
|  | ID1-R | GTAGGTGTGCAGAGAGGAGC |
|  | ID2-F | TCGAAGAGCTGCAATGTCCA |
|  | ID2-R | TACGTGCCACAGTGGAACAG |
